# *Toxoplasma gondii* and *Neospora caninum* in farm-reared ostriches (*Struthio camelus*) in China

**DOI:** 10.1186/s12917-017-1221-2

**Published:** 2017-10-11

**Authors:** Yongjie Feng, Yaoyao Lu, Yinghua Wang, Longxian Zhang, Yurong Yang

**Affiliations:** 1 Laboratory of Veterinary Pathology, College of Animal Science and Veterinary Medicine, Henan Agricultural University, Zhengzhou, 450002 People’s Republic of China; 2 Center for Animal Disease Control and Prevention of Henan Province, Zhengzhou, 450002 People’s Republic of China

**Keywords:** Antibody, Cysts, DNA, Epidemiology, Modified agglutination test

## Abstract

**Background:**

The parasites *Toxoplasma gondii* (*T. gondii*) and *Neospora caninum* (*N. caninum*) are globally distributed; they infect warm-blooded animals, including many avian species. The aim of this study was to evaluate the presence of these parasites in ostriches from central China. In total, 402 ostrich (*Struthio camelus*) samples (293 hearts, 77 brains, and 32 serum) from slaughterhouses of the Henan Province and Hebei Province were collected. The heart juice (*n* = 283) and serum samples (*n* = 32) were tested for antibodies to *T. gondii* using the modified agglutination test (MAT). Hematoxylin and eosin (H&E) staining, immunohistochemical (IHC) staining, and the polymerase chain reaction were used to examine the cysts and DNA of *T. gondii* and *N. caninum* parasites, respectively.

**Results:**

Antibodies to *T. gondii* were detected in 6.4% (20/315) (cut-off, 25). No cysts or DNA of *T. gondii* or *N. caninum* were observed in any of the 293 hearts and 77 brains.

**Conclusion:**

The results showed a low prevalence of *T. gondii* antibody in ostriches, compared to that in the other animals. *N. caninum* occurs at low to negligible frequencies in ostriches from China. This is the first report on screening ostriches in China for *T. gondii* antibodies.

## Background

*Toxoplasma gondii* is an obligate cyst-forming parasite known to infect many species of warm-blooded animals, including ostrich [1, 2]. Infection with *T. gondii* is usually asymptomatic, but it can cause severe illness in both humans and animals with weakened immune systems, and in the case of pregnancy [3]. *Neospora caninum* is a parasite similar to *T. gondii* in many respects as it causes abortion in cattle and paralysis in dogs [4, 5].

The breeding of ostriches for eggs, feathers, skin and meat is growing in China and its meat, in particular, is low in fat and highly palatable. The prevalence of *T. gondii* in free-range chickens is a good indicator of the presence of oocysts in the environment [6], and ostrich is much like free-range chickens that feed directly from the ground. It was speculated that *T. gondii* and *N. caninum* infection in ostriches may assess the presence of oocysts in the local soil and environment. Furthermore, the consumption of undercooked ostrich meat containing *T. gondii* cysts might be a source of infection to consumers. Although the prevalence of *T. gondii* in ostriches has been reported in various countries [7–13], no research studies on *T. gondii* infection in ostriches from China have been conducted to date. Recently, the first report of isolation of *T. gondii* from ostriches was published [2]. The aim of this study was to determine the presence of *T. gondii* and *N. caninum* in farm-raised ostriches from China and attempt to isolate *T. gondii*.

## Methods

### Sample collection

A total of 402 ostrich (*Struthio camelus*) samples (283 fresh hearts, 77 fresh brains, 32 fresh sera, and 10 formalin-fixed hearts) from slaughterhouses in two provinces (Henan Province: 165 hearts, 77 brains, 32 sera and 10 formalin-fixed hearts samples; Hebei Province: 118 hearts) were collected between 2012 and 2015 (Table 1). All ostriches was above 10 months old, however the sex was unknown. The provinces of Henan (31.38° to 36.37°N and 110.35° to 116.65°E) and Hebei (36.08° to 42.67° N and 113.45° to 119.83°E) are located in central China. Unfrozen and fresh samples were transported to the Laboratory of Veterinary Pathology, Henan Agricultural University in cooler boxes. The ostriches were bred in paddocks and fed from the ground with greens and nutritious additives. Heart juice from 283 hearts was collected and centrifuged (1000 *g*, 5 min) and then assayed for antibodies against *T. gondii*. A total of 315 samples (283 heart juice and 32 sera) were screened for *T. gondii* antibodies. The samples were kept in a refrigerator with a temperature range of 2 °C to 4 °C until analysis (within 1 week).

**Table 1:** Samples from Ostriches raised on farms in Henan Province and Hebei Province (*n* = 402)

Location	Collection time	Samples No.	Positive No. in different titers				Total positive No. and rate (%)	Isolation obtained by mice from samples^a^
			25	50	100	200		
Hebei Province	2015 Summer/fall	118 hearts	0	0	0	0	–	0
Henan Province	2015 Spring^b^	31 hearts	1	0	0	0	1/31 (3.2%)	0/1
	2015 Fall^b^	134 hearts 66 brains 32 sera	13 – 6	10 – 6	8 – 6	0	19/166 (11.5%)	0/7
	2012–2014	10 hearts^c^ 11 brains^c^	–				–	0
Total	2012–2015	293 hearts 77 brains 32 sera	20	16	14	0	20/315 (6.4%)	0/8

^a^Number of antibody-positive groups /Number of inoculated-groups

^b^ *P*-value >0.05 by two-tailed chi-square tests for season factor in Henan Province

^c^formalin fixed samples

### Screening of ostriches body fluids for *T. gondii* antibodies

Using the modified agglutination test [14] at a dilution of 1:25, 1:50, 1:100 and 1:200, 315 body fluid samples (283 heart juice and 32 sera) were screened for *T. gondii* antibodies. *T. gondii-*positive serum from mice was used as a reference; negative serum and blank were performed in each plate. Whole formalin-treated *T. gondii* tachyzoite antigen was obtained from the Kerafast Company (Boston, MA, USA. Catalog No. EH2002). Serum dilution of 1:25 is effective for assessing the *T. gondii* antibody, which has been reported in ostriches and other animals [7, 8].

### Histopathological examination of ostrich heart and brain tissues for *T. gondii* and *N. caninum*

A total of 370 ostrich tissue samples (293 hearts and 77 brains) were respectively cut into three pieces (1.0 × 1.0 × 0.3 cm), embedded in paraffin, and sectioned at a thickness of 5 μm. These tissue sections were then stained with hematoxylin and eosin (H&E). Immunohistochemical (IHC) staining was used to verify suspected samples by rabbit polyclonal *T. gondii* antibody and mouse polyclonal *N. caninum* antibody. *T. gondii-*positive tissues or *N. caninum-*positive tissues from mice were used as reference; negative serum and blank were performed in each batch. The sections were examined with an Olympus CX21 optical microscope to search for cysts and determine the infection of *T. gondii* or *N. caninum*.

### Isolation of viable *T. gondii* from ostrich hearts by bioassay in mice

Ostrich hearts that tested positive for *T. gondii* antibodies were bioassayed in mice individually (*n* = 8); sheep hearts containing *T. gondii* cysts were used as controls. Myocardia (50 g) from each heart were homogenized, digested in pepsin, and inoculated subcutaneously into 5 outbred *Kunming* mice, which were given water supplemented with dexamethasone phosphate (10 μg/ml) 3 days before inoculation [3]. The remaining pepsin-digested myocardial samples were stored in 1.5 mL cryotubes at −20 °C until further analysis. Clinical signs in these mice were observed daily. The mice were bled and sacrificed at 64 days post inoculation. Then the sera were diluted 1:25 and 1:200 to test for *T. gondii* antibodies; in addition, squash preparations of the brain were microscopically examined for *T. gondii* cysts. The mouse brains were then inoculated into new groups of mice.

### PCR identification of *T. gondii* and *N. caninum* in ostrich heart and brain tissues

DNA was extracted from all tissue samples (293 hearts, 77 brains, and 8 pepsin -digested sediments of the myocardium) using a commercial DNA extraction kit (Tiangen Biotec Company, Beijing, China, DP304). PCR was used to detect the DNA of *T. gondii* (TOX5/TOX8) in amplified fragments of 450 bp [15] and *N. caninum* (NP6/NP21) in fragments of 337 bp [16]. The DNA isolated from *T. gondii* (CT1 strain) or *N. caninum* (NC1 strain) was used as a reference for PCR.

### Statistical analysis

Statistical analysis was performed using GraphPad Prism 4.0 (GraphPad Software Inc., San Diego, CA, USA). Data were analyzed by using the chi-square test or Fisher’s exact test. *P* < 0.05 was considered statistically significant.

## Results and discussion

### *T. gondii* antibody testing

*T. gondii* antibodies have been previously detected in the meat juice of pig, sheep, and cattle [17, 18]. In the present study, by using MAT, *T. gondii* antibodies were found in 6.4% (20/315) of the ostriches, with titers of 25 in 20, titers of 50 in 16, titers of 100 in 14 (8 hearts and 6 sera), and none showed a titer >200. The seroepidemiology of *T. gondii* was 10.2% (20/197: 165 hearts, 32 sera) in Henan Province. None of the samples from Hebei Province (118 hearts) were positive (Table 1). Hebei is located north of Henan, drier and colder. The climate of Hebei Province may contribute to the negative toxoplasmosis results involving ostriches from the region. In Henan Province, 11.5% (19/166) of the ostrich samples that were collected in the fall were seropositive for *T. gondii*, whereas 3.2% (1/31) of those collected in the spring were seropositive. The risk of *T. gondii* infection in the fall was thus higher compared to that in the spring, with an odds ratio of 3.878 (95% CI, 0.4995–30.10). However, this difference was not statistically significant (*P* > 0.05). This finding suggests that ostriches from Henan Province had been in contact with *T. gondii* oocysts from cats or from soil, water, or food. Ostriches feed from the ground, are raised much like free-range chickens and have a relatively free activity field. In Henan Province, the seroprevalence of *T. gondii* in free-range chickens was 18.9% (132/700) [19]; in cats, it was 52.3% (102/195) and 51.6% (16/31) [20, 21]; 12.7% (99/779) and 29.3% (83/283) in sheep [22, 23], 23.7% (627/2642) in pig [24]. Compared with these reports, seroprevalence of *T. gondii* was lower among farm-reared ostriches (10.2%, 20/197) in the same location. This result is consistent with other reports. A low prevalence of *T. gondii* antibodies in ostriches has been reported in other countries. The seroprevalence of *T. gondii* was about 74% to 80% among backyard chickens in Brazil [25, 26], but lower among ostriches (about 11% to 17%) [2, 9, 10] (Table 2). A summary of the prevalence of *T. gondii* in ostriches in various countries is shown in Table 2. Prevalence varies from 1% to 48%, depending on the country. In conclusion, this is the first report to show the prevalence of *T. gondii* in ostriches from China.

**Table 2:** Prevalence of *Toxoplasma gondii* infection in ostriches

Country	No. of samples received	No. of seropositive (%)	Serologic test (cut-off titer)	References
Zimbabwe	50	24 (48%)	MAT (25)	[8]
Canada	973	28 (3%)	MAT (25)	[7]
Brazil	46	8 (17%)	MAT (16)	[9]
	195	28 (14%)	MAT (16)	[10]
	344	38 (11%)	MAT (8)	[2]
Spain	117	1 (1%)	MAT (25)	[11]
Egypt	120	15 (13%)	MAT (25)	[12]
Iran	28	6 (21%)	ELISA	[13]
China	315	20 (6%)	MAT (25)	This study

### *T. gondii* isolation, *T. gondii* and *N. caninum* morphological and molecular assays

Viable *T. gondii* had been isolated from ostrich brains, thereby serving as direct evidence that ostriches are intermediate hosts [2]. Isolation of *T. gondii* from the chicken heart or brain by bioassay in mice has been shown to be effective [3, 27]. A previous study has shown that the density of *T. gondii* cysts in the heart is higher than that in muscle or brain of chickens [27]. Eight ostrich hearts were bioassayed in immunosuppressed *Kunming* mice in an effort to isolate *T. gondii*, but this was unsuccessful. Furthermore, H&E or IHC staining did not detect any tissue cysts of *T. gondii* or *N. caninum*, and no positive DNA of *T. gondii* or *N. caninum* was detected by PCR in a total of 370 ostrich tissue samples and 8 pepsin-digested liquids from myocardia. These results may be explained by (1) the relative low density of *T. gondii* cysts in ostriches from China or (2) false positive on the MAT. Antigen from whole formalin-treated tachyzoites was used in the MAT. The MAT is highly sensitive and has been extensively used to test *T. gondii* antibodies in many animals and birds, including ostriches. The accuracy of the MAT has been validated in ostriches because 14/38 (36.8%) isolation has been achieved [2]. However, we can not rule out the possibility that *T. gondii* antigen cross-reacts with other parasites (*Hammondia hammondi*) [28].

Some birds (chickens, pigeons, sparrows) have been shown to be intermediate hosts for *N. caninum* [29]*.* However, a recent literature search has found no study on *N. caninum* in ostriches. Ostriches have not been proven to be intermediate hosts for *N. caninum*. An examination of tissue sections was the most straightforward way of observing the parasites and lesions by light microscopy. Mineo found that no serological positivity of *N. caninum* was observed in 294 samples of serum. However, *N. caninum* cysts were found in Psittaciformes muscle by tissue section [30]. PCR is a sensitive method for detecting parasites. The DNA of *N. caninum* in the heart and brain of many animals has detected by PCR, particularly in the brains [31]. The absence of *N. caninum* from these ostrich samples suggests that this parasite occurs at low to negligible frequencies in ostriches from China. Further investigations using samples from different areas may facilitate in better understanding the process of neosporosis in ostriches.

## Conclusions

The results of the present study indicate that *T. gondii* has a lower prevalence in ostriches compared to other animals, the cause of this difference is unknown. However, consumers are precautions of eating raw or undercooked ostrich meat to avoid being infected with *T. gondii*. *N. caninum* occurs at low to negligible frequencies in ostriches from China. The establishment of differences in prevalence rates between ostrich and other birds may assist in the design of preventive and control measures against toxoplasmosis.

## Abbreviations

H&E: Hematoxylin and eosin staining
IHC: Immunohistochemistry
MAT: Modified agglutination test
PCR: Polymerase chain reaction
